# Marriage in the Melting Pot: An Evolutionary Approach to European Ancestry, Homogamy, and Fertility in the United States

**DOI:** 10.3389/fpsyg.2022.614003

**Published:** 2022-07-11

**Authors:** Alexander Schahbasi, Susanne Huber, Martin Fieder

**Affiliations:** ^1^Department of Evolutionary Anthropology, Faculty of Life Sciences, HEAS - Network of Human Evolution and Archeological Sciences, University of Vienna, Vienna, Austria; ^2^Erlangen Centre for Islam and Law in Europe, Friedrich-Alexander-Universität Erlangen-Nürnberg, Erlangen, Germany; ^3^Forschungszentrum “Religion and Transformation in Contemporary Society”, Universität Wien, Vienna, Austria

**Keywords:** evolution, marriage, homogamy, fertility, social cohesion, heritability

## Abstract

To understand marriage patterns, homogamy, and fertility of women of European ancestry in the United States from an evolutionary perspective, we investigated if a prevalence of ancestral homogamy exists, the factors influencing a female preference for an ancestral homogamous vs. heterogamous marriage, and if ancestral homogamous vs. heterogamous marriages have an impact on fertility. Furthermore, we aim to determine the heritability of homogamous vs. heterogamous marriage behavior. We used the census data of 369,121 women in the United States married only once and aged between 46 and 60 years, provided by IPUMS USA (https://usa.ipums.org/usa/). We used linear mixed models to determine the association between the probability of a homogamous vs. heterogamous marriage and the individual fertility of women. We aimed to estimate the heritability (genetics and parental environment) of marriage behavior using a linear mixed model. We found that ancestral heterogamous marriages are more frequent compared to homogamous marriages, but only if all ancestry groups are included. If ancestry is aggregated, homogamous marriages are more frequent compared to heterogamous marriages. Most of the variance (up to 27%) in inter-ancestry marriage and fertility (up to 12%) is explained by ancestry *per se*, followed by the ratio of individuals of a certain ancestral background in a county (∼6%), indicating a frequency depending selection into marriage: the more individuals of a certain ancestry live in a county, the lower is the tendency to marry someone of a different ancestral background. Furthermore, we found that about 12% (depending to some extent on the clustering) of the marriage behavior is heritable. Being in a homogamous marriage and the income of the spouse are both significantly positively associated with the number of children women have and the probability that women have at least one child, albeit explaining only a very low proportion of the overall variance. The most important factor (in terms of variance explained) for being in an ancestral homogamous vs. heterogamous marriage, for the number of children, and for childlessness is the ancestry of the women. Most children are born to women of Irish, French, and Norwegian ancestry (Irish X̄: 3.24, French X̄: 3.21, and Norwegian X̄: 3.18), the lowest number of children is to women of Latvian, Rumanian, and Russian ancestry (Latvian X̄: 2.26, Rumanian X̄: 2.19, and Russian X̄: 2.35). Albeit, we are not able to distinguish the genetic and social heritability on the basis of our data, only a small heritability for in-group vs. out-group marriage behavior is indicated (∼12% of variance explained).

## Introduction

When investigating marriage patterns, homogamy and fertility with regard to the long-term implications of migration the findings in the field of archeological genetics – reviewed in Reich (2018) – are of particular relevance as they indicate the enormous genetic impact of migration and admixture in shaping the human genome. These findings on the migration flows and global dispersal of Homo sapiens allow for the classification of migration processes and subsequent admixture as an inherent trait of the human species. Migration flows induced processes of human interactions taking place on a continuous spectrum, ranging from intermarriage/admixture to hostile/violent encounters at its extremes. Understanding these processes may help to comprehend long-term social cohesion in the context of migration. From the perspective of kin selection (Hamilton, 1964), we argued that long-term social cohesion between groups of different cultural, ethnic, and religious origins is ensured if genetic bonds transcend group divisions (Fieder et al., 2020). At the same time, we are aware that humans have a strong tendency toward homogamy, which has been demonstrated by several factors, e.g., body height (Stulp et al., 2013, 2017), religion (reviewed in Fieder and Huber, 2016), political attitudes, and ethnicity (Blackwell and Lichter, 2004; Fu and Heaton, 2008). Particularly the tendency to engage in ethnically and religiously homogamous marriages is an opposing trend to intermarriage (Fieder and Huber, 2016; Huber and Fieder, 2018; Schahbasi et al., 2020). Furthermore, educational attainment is an increasingly important factor in the context of homogamous mating, particularly for the lower and higher educated strata of society (Blackwell and Lichter, 2004; Fieder et al., 2011).

It has to be taken into consideration that within short-term and long-term mating strategies (Gangestad and Simpson, 2000), different parameters are of relevance. As in long-term partners, factors such as paternal care and agreeability play a greater role; this may indicate a preference for homogamy. From an evolutionary point of view, the decisive factor is if assortative mating within cultural, ethnic, or religious groups leads to any selective advantages, i.e., whether assortative mating leads to an increase in the number of children. Assortative mating and the prevalence of homogamy have often been documented, but a potential correlation between homogamy and reproduction has only been investigated for a few traits so far. Studies find that educational homogamy is particularly associated with a lower prevalence of childlessness (Huber and Fieder, 2011, 2016; Van Bavel, 2012), and religious homogamy is positively associated with both fertility and having at least one child (Fieder and Huber, 2016). Moreover, religious homogamy may compensate for ethnic heterogamy in terms of reproduction and vice versa (Huber and Fieder, 2018). It has also been shown that increasing height differences between spouses may increase the necessity of a cesarean section (Stulp et al., 2011) and thus may also been a selective force in the past.

It has been theorized that humans may detect genetic similarities on the basis of phenotypic traits and mate assortatively along with these traits and such behavior may enhance fitness (Rushton, 1985; Salter, 2002). Marrying someone who is genetically closer may bring reproductive benefits as has been demonstrated on the basis of data from Iceland: the average number of offspring decreases with genetic relatedness from second-order cousins (Helgason et al., 2008). Thus, during our evolutionary past, moderate inbreeding may have led to reproductive benefits (Fox, 2015) while leading to higher homozygosity and an association with health, intellectual, and other related problems (Clark et al., 2019). During our evolutionary past, moderate inbreeding may have led to reproductive benefits (Fox, 2015) while also leading to higher homozygosity and the related physical and intellectual issues (Clark et al., 2019). Luo (2017) summarized four points that may foster homogamy and lead to more similar mates: (i) couple similarity due to active choice: assortative mating may increase altruism due to inclusive fitness (Hamilton, 1964) and if spouses are more similar in terms of shared genetics, genetic similarity between parents and offspring may exceed 50%; (ii) also market operations may lead to assortment: for instance, if someone desires a partner of high mate value (e.g., attractiveness or status), but his/her mate value is low, he/she has to choose a more similar partner; (iii) social homogamy: if someone lives in an area where people of the same religion or ancestry live, the probability is higher so that she/he also will marry someone from their own group, and (iv) convergence: spouses may grow more similar in the course of time, e.g., they may become more similar in appearance (Zajonc et al., 1987).

Based on these deliberations, we aim to investigate homogamous vs. heterogamous marriage patterns and the influence of homogamy/heterogamy on the reproduction of women in the United States. Data from the census of 1980 were used for the analysis as only this census covered all the variables needed for a stringent investigation. Based on previous studies, we assume that the share of individuals from a certain ancestry in a geographical region, education, and income influences the willingness to marry heterogamous (Blau et al., 1982; Fieder et al., 2020) and if assortative mating is occurring by the chance or not. Furthermore, findings suggest (Huber and Fieder, 2011, 2016, 2018; Fieder and Huber, 2016) that in homogamous relationships, the number of children is increasing and childlessness is decreasing, leading to fitness benefits. In detail we investigated (i) if a prevalence of ancestral homogamy exists; (ii) which factors influence the preference for an ancestral homogamous vs. heterogamous marriage; (iii) if ancestral homogamous vs. heterogamous marriages influence fertility (measured in the number of children and childlessness); and (iv) the inherited component of the tendency to marry homogamous or heterogamous. To ascertain that not only first-generation migrants may have been responsible for the found associations we also conducted the same analysis excluding all individuals not born in the United States.

## Materials and Methods

To investigate the prevalence of homogamy, the number of children and childlessness according to the ancestry of United States women and their spouses we used the following data sets: (i) data set 1: the census records of 369,121 women married only once aged between 46 and 60 years (almost completed or completed reproduction) and their spouses and (ii) data set 2: the census records of 2,721 women aged between 16 and 35 years, married only once, their spouses and their parents, all living in the same household. Both data sets have been extracted from the United States census of 1980, provided by IPUMS USA^1^ (Ruggles et al., 2022). We only included individuals of European ancestry in our analysis, as interracial marriages are comparably rare, particularly among the largest groups–White and Black/African Americans (over 99% in Whites and Blacks–Supplementary Table 1). As ancestry in census data sets is not an unambiguous category and original ancestry groups (Table 1) largely differ in size, we aggregated the ancestry variable in two steps to larger clusters omitting only small groups and mixed ancestry: (i) moderately clustered; 15 ancestry with no cluster smaller than 200 individuals in total 315, 094 women (Table 2); and (ii) substantially clustered–only the “principal big ancestry” five clusters not smaller than 20,000 individuals in total 283,737 women (Table 3). We made all calculations of the number of children, childlessness, and the probability of heterogamous vs. homogamous marriages separately on the basis of these three consecutive clustered data sets. Furthermore, we calculated for each ancestry group a “null model,” “odds ratios of homogamy” according to Lieberson and Waters (1988) and Rosenfeld (2002); hence, a theoretical model where homogamy has no influence and mating occurs only by random independent from preferences and actual geographical distributions.

**TABLE 1:** “Raw ancestry” of the women in our sample, counts, and percentages.

	*N*	%
English	94020	25.5
German (1980)	76668	20.8
Irish	48740	13.2
Italian (1980)	27009	7.3
French (1980)	17509	4.7
Polish	17292	4.7
Scottish	16852	4.6
Dutch	8882	2.4
Russian	6753	1.8
Swedish	6738	1.8
Norwegian	6078	1.6
Hungarian	3216	0.9
English-Irish-Scotch	2884	0.8
Czechoslovakian	2859	0.8
Spanish	2818	0.8
English-German-Irish	2542	0.7
Danish	2441	0.7
Greek	2315	0.6
Austrian	2163	0.6
Portuguese	2024	0.5
Slovak	1982	0.5
Ukrainian (1980)	1867	0.5
Swiss	1672	0.5
German-Irish-Scotch	1544	0.4
Lithuanian	1526	0.4
French Canadian	1502	0.4
English-French-German	1142	0.3
Finnish	983	0.3
English-French-Irish	929	0.3
German-French-Irish	784	0.2
Rumanian (1980)	726	0.2
Canadian	716	0.2
Belgian	629	0.2
Croatian	571	0.2
Dutch-Irish-Scotch	391	0.1
Dutch-German-Irish	338	0.1
Slovene	338	0.1
Serbian (1980)	244	0.1
Spanish American	217	0.1
Latvian	203	0.1
English-German-Swedish	102	0
Albanian	99	0
Dutch-French-Irish	95	0
Luxembourg	90	0
English-Scotch-Welsh	83	0
Icelander	74	0
German-Irish-Italian	72	0
Australian	72	0
Maltese	67	0
German-Irish-Swedish	62	0
Estonian	59	0
Bulgarian	54	0
Macedonian	47	0
Basque (1980)	38	0

**TABLE 2:** Ancestry moderately clustered.

	*N*	%
1. United Kingdom, British:: English, Scottish, Australian, Canada English	107092	33.99
2. Irish	47387	15.04
3. Scandinavian: Danish, Swedish, Fin, Norwegian, Icelander	31170	9.89
4. German Speaking: Austria, Germany	74308	23.58
5. Dutch	8320	2.64
6. French, French Canadian	2101	0.67
7. Italian	23780	7.55
8. Iberian: Spanish, Portuguese, Spanish American	3036	0.96
9. Croatian, Slovenian	835	0.27
10. South Slavic, Orthodox	274	0.09
11. Greek	1523	0.48
12. Eastern Slavic: Ukrainian, Russian	7568	2.40
13. Middle European Slavic: Czechoslovakian, Slovakian	4495	1.43
14. Polish	2655	0.84
15. Hungarian	550	0.17

**TABLE 3:** Ancestry substantially clustered.

	*N*	%
1. United Kingdom, British: English, Scottish, Australian, Canada English	107092	37.7
2. German Speaking: Austria, Germany	74308	26.2
3. Irish	47387	16.7
4. Scandinavian: Danish, Swedish, Fin, Norwegian, Icelander	31170	11.0
5. Italian	23780	8.4

The odds ratio for endogamy is calculated as (N1/N2)/(N3/N4), illustrated here in the example on the basis of English ancestral homogamy: N1 is the number of English men married to English women (N1 = 72021), N2 is the number of English men married to non-English women (N2 = 63911), N3 is the number of non-English men married to English women (N3 = 61629), and N4 is the number of non-English men married to non-English women (N4 = 833,491). (72021/63911)/(61629/833491) = 15.24, hence individuals of English ancestry tend to marry 15.24 more frequently homogamous as predicted by chance if there would be no assortative mating in place.

We further used data set 2 to investigate the proportion of the additive genetic heritability and the proportion of the paternal social heritability of marrying homogamous vs. heterogamous according to ancestry, using a linear mixed model. We included the following variables in the analyses: woman’s age, her age at first marriage, woman’s own education (encoded in 21 steps, further used as a continuous variable–Supplementary Table 2), the total yearly income of the woman in a year and of her spouse, the ratio of persons of a certain ancestry in a United States county (“ratio ancestry county,” calculated as the number of habitants of a certain ancestry in a county/total number of inhabitants of a county), number of children born to a woman and her childlessness (encoded: 0 = childless, 1 = one or more children), if a women is in an ancestral homogamous or heterogamous marriage according to the three different aggregations of ancestry clusters (0 = heterogamous and 1 = homogamous, HomHetGam), and ancestry of the women (encoded as described in Supplementary Table 1) and for data set 2 only if the women’s parents are in an ancestral homogamous vs. heterogamous marriage (0 = heterogamous and 1 = homogamous, ParentalHomHetGam).

### HomHetGam, Number of Children and Childlessness

On the basis of data set 1, we calculated the following three separate general linear mixed models for all 3 different aggregations of ancestry in sum nine models (no aggregation, moderate aggregated 15 clusters, and strongly aggregated five clusters): (i) age, age at first marriage, education, total income, the total income of spouse, and ratio ancestry county, regressing on Homogamous/Heterogamous (HomHetGam) on the basis of a binomial error structure; (ii) HomHetGam, age, age at first marriage, education, total income, total income spouse, and ratio ancestry county regressing on number of children on the basis of a Poisson error structure, and (iii) HomHetGam, age, age at first marriage, education, total income, total income spouse, ratio ancestry county regressing on childlessness on the basis of a binomial error structure. In all three models, the ancestry of a woman was included as a random factor. Mixed models were calculated in R library (MASS), and function glmmPQL.

### Genetic and Social Heritability

de Villemereuil et al. (2016) published an article showing a new methodological approach for inference of quantitative genetic parameters, also on the basis of non-normal distributions of phenotypes (binary, Poisson) in the statistical framework of the generalized linear mixed models (glmm). Using the glmm (so-called animal model), it is possible to estimate additive genetic variances and covariances by including pedigree data for the investigated phenotype for relatives. In our case, the phenotype (ancestral homogamy–binary trait) of an individual is regressed on the corresponding binary phenotype of the father and the mother (random terms). In the case of homogamy, the phenotype “homogamy” is of course the same for the father and the mother (hence redundantly used). With this approach in difference to “twin models,” we are not able to separate between additive genetics and a common environment.

Thus, on the basis of data set 2, we calculated the following linear mixed model^2^ (de Villemereuil et al., 2016): HomHetGam regressing on the random factor HomHetGam if (1 if HomHetGam is homogamous and 0 if HomHetGam is heterogamous) controlling for age and education as fixed factors, on the basis of a binomial error structure [i.e., HomHetGam = *age + education + random* (HomHetGam)]. We calculated the general linear mixed model using R, library lme4, function glmer. “Heritability” was calculated by the function QGparams from the R library QGglmm. We used the QGglmm packages as these packages enable the calculation of heritability for mixed models, not only linear mixed models, but also general linear mixed models, e.g., for a binary phenotype such as being in a homogamous relationship (de Villemereuil et al., 2016).

We further provided a table with the raw correlations among the variables in our analysis using person correlation for the continuous and countable variables and tetrachoric correlation for the binary variables (Supplementary Table 3).

## Results

### HomHetGam

We found that when using only the raw, not aggregated ancestry categories, ancestral heterogamous marriages are more frequent (56.5%) compared to homogamous marriages (43.5%). In the aggregated clusters, homogamy becomes more frequent and makes up the majority of the cases (Table 4).

**TABLE 4:** Frequency of HomHetGam, according to aggregation in clusters.

	% Heterogamous	% Homogamous	Total number cases
Original ancestry groups	56.5	43.5	369,121
Ancestry cluster	49.6	50.4	345,356
Big ancestry clusters	45.3	54.7	253,358

There is clearly non-random mating in ancestry ongoing; hence, the rate of homogamy is much higher as predicted by random mating only, indicated by the odds ratio (null model) independent from clustering (Supplementary Tables 4–6). In the non-clustered data, the odds ratio ranges from 10 times more frequent homogamous marriages for German ancestry to almost 15.000 more frequent homogamous marriages among individuals of Cuban ancestry compared to simple random mating without any preference for homogamy (Supplementary Table 4). The span in the difference of odds ratio is less pronounced if the data are clustered (Supplementary Tables 5, 6) but still is enormous; hence, ancestral homogamy exceeds by far random mating and heterogamy is by far less frequent than expected by random mating only.

Further age and the ratio of individuals of a certain ancestry in a county are significantly positively associated with being in a homogamous marriage, whereas age at first marriage and spouse’s income and education are significantly negatively associated with being in a homogamous marriage. Beta-values vary with aggregation in clusters, but overall the direction of effects remains the same. Most of the variance (26.6, 11.55, and 11.50% decreasing according to clustering) depending on the aggregation in HomHetGam is explained by the ancestry of women (random factor); hence, the ancestral background largely determines HomHetGam. From the fixed factors, most of the variance is explained by the ratio of individuals of a certain ancestral background in a county (6%), varying with aggregation (6.4, 6.8, and 5.35%): the more individuals of a certain ancestry live in a county, the lower is the tendency to marry someone of a different ancestral background. Second, the highest proportion (1.3%) of the fixed factors in the variance of HomHetGam is explained by the highest education, also varying by aggregation (1.1, 0.8, and 0.9%–Table 5). The other explaining variables depict only a small proportion of the variance, and most variance is explained by the income of the spouse (Table 5).

**TABLE 5:** Being in an ancestral heterogamous relationship vs. a homogamous relationship regressing on the ratio of the same ancestral group in a county, age, age at first marriage, education, income, spouse’s income, R^2^ for each explaining variable, the sum of R^2^ of all explaining variables, and R^2^ of the random factor ancestry.

	(A) Original ancestry groups	R^2^ %	(B) Ancestry cluster	R^2^ %	(C) Big ancestry clusters	R^2^ %
Ratio ancestry county	1.05***	6.4	0.99***	6.8	0.86***	5.35
Age	0.04***	0.03	0.04***	0.03	0.03	0.02
Age first marriage	–0.06***	0.11	–0.05***	0.09	–0.02*	0.06
Education	–0.37***	1.1	–0.3***	0.8	–0.31***	0.9
Income	–0.01^NS^	0.07	–0.01^NS^	0.06	0^NS^	0.05
Income spouse	–0.09***	0.32	–0.06***	0.24	–0.07***	0.27
Sum R^2^ all explaining variables		**8.03**		**8.02**		**6.65**
R^2^ % random ancestry		**26.6**		**11.55**		**11.5**

Bold values indicate the sum of variance explained by the explaining factors and by the random factors in the linear mixed model. *P < 0.05, **P < 0.01, and ***P < 0.001.

### Number of Children

We found that age, age at first marriage, and women’s own income are significantly negatively associated with a woman’s number of children (Table 6). Being in an ancestral homogamous marriage, the income of the spouse is significantly positively associated with her number of children. Education is in no significant association, respectively, only a marginally significant negative association in the case of the most aggregated ancestral cluster, with the number of children a woman has. Albeit estimates vary in size, a comparable pattern can be found across aggregated clusters. The highest proportion of variance is explained by the random factor “ancestry,” but decreases with aggregation. (26.6, 11.55, and 11.50%). *l*HomHetGam explains not more than 0.1% of the variance in the number of children. In detail, most children are born to women of Irish, French, and Norwegian ancestry (Irish X̄: 3.24, French X̄: 3.21, and Norwegian X̄: 3.18), and the lowest number of children to women of Latvian, Rumanian, and Russian ancestry (Latvian X̄: 2.26, Rumanian X̄: 2.19, and Russian X̄: 2.35).

**TABLE 6:** General linear mixed models of age, age at first marriage, education, income, the income of the spouse, the ratio of the own ancestry group in a county, and HOMHETGAM, regressing on woman’s number of children on the basis of a Poisson error structure, with ancestry as a random factor.

	(A) Original ancestry groups	(B) Ancestry cluster	(C) Big ancestry clusters
HomHetGam	0.0071*** (R^2^ % 0.1)	0.0078*** (R^2^ % 0.1)	0.0074*** (R^2^ % 0.1)
Age	–0.019***	–0.018***	–0.018***
Age first marriage	–0.09***	–0.089***	–0.086***
Education	0.0008^NS^	–0.001^NS^	–0.0013
Income	–0.028***	–0.028***	–0.027
Income spouse	0.0079***	0.0076***	0.008***
Sum R^2^ % all explaining variables	**9.27**	**9.27**	**9.27**
R^2^ % random Ancestry	**11.8**	**3.6**	**2.0**

Separate models for clusters of aggregation in rows. In columns A, B, and C, the separate models for the different clusters are shown. Bold values indicate the sum of variance explained by the explaining factors and by the random factors in the linear mixed model. *P < 0.05, **P < 0.01, and ***P < 0.001.

### Childlessness

We found that age at first marriage, a women’s own income is significantly negatively associated with her probability of having at least one child, whereas being in a homogamous marriage, education and the income of the spouse are significantly positively associated with having at least one child. As is the case in the other models, most of the variance is explained by the random factor “ancestry,” but varies slightly according to the aggregation of ancestry (13.6 13.38, and 12.99%). HomHetGam explains not more than 0.1% of the variance; all factors together explain around 13% of the variance, differing according to aggregation (Table 7).

**TABLE 7:** General linear mixed model of HOMHETGAM, age, age at first marriage, education, income, the income of the spouse, and the ratio of the own ancestry group in a county regressing on childlessness (encoded as 0 = childless, 1 = one or more children) on the basis of a binomial error structure, with ancestry as a random factor.

	(A) Original ancestry groups	(B) Ancestry cluster	(C) Big ancestry clusters
HomHetGam	0.1717*** (R^2^ % 0.1)	0.1854*** (R^2^ % 0.1)	0.1497*** (R^2^ % 0.1)
Age	–0.238***	–0.242***	–0.262***
Age first marriage	–2.477***	–2.469***	–2.436***
Education	0.2331***	0.2074***	0.205***
Income	–0.624***	–0.628***	–0.638***
Income spouse	0.59	0.593	0.5911
Sum R^2^ % all explaining variables	**13.4**	**13.23**	**12.9**
R^2^ % random ancestry	**13.6**	**13.38**	**12.99**

Separate models for clusters of aggregation in rows. In columns A, B, and C, the separate models for the different clusters are shown. Bold values indicate the sum of variance explained by the explaining factors and by the random factors in the linear mixed model. *P < 0.05, **P < 0.01, and ***P < 0.001.

A limitation is that we do not know how far the ancestry dates back, and we can only assume that the longer migration dates back, the weaker the effects of ancestry should be. To ascertain that not only first-generation migrants may have been responsible for the found associations, we conducted the same analysis excluding all individuals not born in the United States. The principal patterns of estimates, significances, and variance explained remained unchanged, indicating that “ancestry effects” are persistent and not restricted to first-generation migrants only (Table 8).

**TABLE 8:** General linear mixed model of (a) HomHetGam regressing on age, age at first marriage, education, income, the income of the spouse, and the ratio of the own ancestry group in a county on the basis of a binomial error structure and (b) the number of children, and (c) childlessness regressing on HomHetGam, age, age at first marriage, education, income, and the income of spouses on the basis of a Poisson error structure, respectively, a binomial error structure, with ancestry as a random factor; excluding all individuals not born in the United States.

	(a) HomHetGam regressing on	(b) Number of children regressing on	(c) Childlessness regressing on
HomHetGam		0.007***	0.17***
Age	0.041***	–0.019***	–0.238***
Age first marriage	–0.063***	–0.09***	–2.477***
Education	–0.368^NS^	0.001^NS^	0.233***
Income	–0.011***	–0.028***	–0.624***
Income spouse	–0.093***	0.008*	0.59***
Ratio ancestry county	1.047***		

*P < 0.05, **P < 0.01, and ***P < 0.001.

### Genetic and Social Heritability of HomHetGam

Regressing HomHetGam on ParentalHomHetGam by using a general linear mixed model with ParentalHomHetGam as a random factor and age and education as fixed factors, we found that genetic and social inheritance account for 11.18% of the variance in HomHetGam. Our model thus suggests that 11.18% of the marital behavior in terms of marrying someone of the same or different ancestry is explained by genetic and social inheritance from the parents (Table 9). The same calculations on the basis of the aggregated clusters showed comparable results, close to the result of the un-clustered ancestry, with a genetic and social heritability of 13.87% for the moderate clusters and 10.92% for the big clusters.

**TABLE 9:** Estimates of social and genetic heritability by a general linear mixed model of HMM on Parent is Homogamous/Heterogamous (ParentalHomHetGam) controlling for age and education.

Random	Effects:			
	Groups	Name	Variance	Std. Dev.
	sire	(Intercept)	0.1358	0.3685
	dame	(Intercept)	0.2575	0.5075
Number	of	obs:	2721,	groups:
Fixed	Effects:			
	Estimate	Std. Error	*z*-value	*P*
(Intercept)	−0.33722	0.44529	−0.757	0.4489
Age	−0.08247	0.04403	−1.873	0.0611
Education	−0.24272	0.04491	−5.404	*P* < 0.0001
**Heritability estimates**				
va: 0.54	vp: 0.39	mu: −0.34		
mean.obs	var.obs	var.a.obs	h2.obs	
0.4232632	0.2441115	0.02730219	0.1118431	11.18%

## Discussion

Ancestry certainly did still play a role in mating behavior in the United States; thus, homogamy on descent still has a high prevalence and ancestral homogamous mating has been still much more common than expected if mating is only random, ranging from at least 10 times deviating from random mating up to 15,000 times. Comparable deviations from non-assortative mating have also been found by Rosenberg for ethnic homogamy (Rosenfeld, 2002). This finding may not only be explained by pure preferences but also by different geographical distributions of immigrants in the United States. It would be interesting to see if at least geographical patterns may have already changed or will change in the future due to the stronger shift of mating into the virtual space.

However, the most important explaining factor (in terms of total variance explained) for being in an ancestral homogamous vs. heterogamous marriage (HomHetGam), for the number of children, and for childlessness is the ancestral background; this complies with a decrease from 26 to 11.5% due to aggregation in variance explained in HomHetGam. Around 13% (slightly varying according to aggregation in clusters) of the variance in childlessness and 4.5% (3.6 and 2.0% for the aggregated clusters, respectively) of the variance in the number of children. Hence, ancestry is a very important predictor for ancestral homogamous vs. heterogamous marriages, number of children, and childlessness in our models, independent of clustering and if controlled for various confounding factors. Hence, fertility traditions and cultures seem to be transmitted by ancestry in the United States, as has been already documented (Fernández and Fogli, 2009). To some extent, ancestry may also reflect ethnicity, but on the basis of our data, we are not able to detangle both. However, depending on the clustering, more than 80% of the variance remains unexplained by the variables in our model. In the case of large data sets, it happens rarely that more than 30% of the variance is explained by the surveyed variables (Fieder and Huber, 2022).

Indeed, frequency-dependent selection, in terms of regional social homogamy, plays a role in the sample: individuals of a certain ancestry who live in a shared geographic area have a higher probability of being socially (ancestrally) homogamous. As it is simpler to find a spouse of the same ancestry, the chance of homogamous marriages increases (Luo, 2017). It is thus reasonable to conclude, that frequency-dependent selection/social homogamy is an important factor for the pattern found, as the ratio of inhabitants of a certain ancestry group in a county positively predicts homogamy and explains ∼6% of the variance in HomHetGam. If more individuals of their own ancestry are at hand for marriage, individuals tend to marry homogamous, a pattern that was previously reported by Thomas (1951) and Blau et al. (1982). Recently, we have also found comparable marriage patterns on the basis of religious denominations in Europe (Fieder et al., 2020).

Apart from ancestry, education positively predicts a homogamous marriage: the higher the level of education of a woman, the higher the probability of a homogamous marriage (but only explaining around 1% of the variance). Her spouse’s income, in contrast, is significantly negatively associated with being in a homogamous marriage. Presumably, this is a result of female mate choice in favor of a wealthy spouse so that spousal income may become more important and thus relaxes mate choice based on ancestry. Interestingly, also the age of first marriage is negatively associated with ancestral homogamy, indicating that the older a woman is at her first marriage, the higher the probability that she marries outside her ancestry.

Ancestry also explains 12% most of the variance of a woman’s number of children but in the case of the non-aggregated ancestry groups only 3.6%, respectively, 2%. The small ancestry groups contributed to the high variance in reproduction and although HomHetGam is significantly positively associated with the number of children, it explains only about 0.1% of the variance. Out of the fixed factors, the age of first marriage explains most of the variance (around 9% in each of the aggregated clusters, data not shown), and it is significantly negatively associated with the number of children; later, a woman marries the fewer children she has, a phenomenon well-known as “postponing” (Schmidt et al., 2012). The age of first marriage is thus a strong predictor of a woman’s number of children.

The patterns of associations between the number of children and SES-indicators were as expected: a woman’s income is significantly negatively associated with the number of children, but her husband’s income is significantly positively associated with the number of children. Education has no significant association with the number of children (respectively in the highly aggregated cluster marginally significant negative), which may be explained by the fact that education leads to a postponing of age at first marriage, which then appears to become the dominating variable in the model. This effect becomes further evident in the model of childlessness: higher education is associated with a lower probability of remaining childless as the age of first marriage is included in the model. But again from the fixed factors, age of first marriage explains 12% (data not shown) of the variance in childlessness, independent of the clustering (data not shown). HomHetGam is significantly positively associated with having at least one child but explains about 0.1% of the variance in having at least one child. Women’s own income is significantly negative and spouses’ income is positively associated with having at least one child. The ratio of an ancestry group in a county is no significant association with childlessness. Ancestry explains around 13% of the variance in childlessness independent from clustering.

In both the number of children and childlessness HH, albeit significant explains only very little of the overall variance, indicating very small effects. We suppose that in our very large sample, even very tiny effects are delectable: effects that may have been much stronger in first-generation immigrants to the United States. It has been demonstrated that for instance, religious homogamy explains up to 10% of the variance in the number of children and childlessness depending on the denomination (Fieder and Huber, in prep)^3^. Hence, homogamy may explain more of the variance in reproduction.

Albeit the effects of HomHetGam on both, the number of children and childlessness, are small in terms of total variance explained, these effects exist whether or not first-generation immigrants are included in the model. Thus, ancestry even from an ancestral rather similar background (European ancestry) seems to influence the reproductive success of women in the United States. This is to some extent comparable to Helgason et al. (2008) and Fox (2015), demonstrating that marrying a genetically more distant individual is associated with a loss in reproductive success. But on the other hand, marrying a genetically too closely related individual is associated with the risk of inbreeding (reviewed in Schahbasi et al., 2020). Additionally, we aimed to estimate the “heritability” of marrying homogamous vs. heterogamous using a generalized linear mixed model (see text footnote 2) (de Villemereuil et al., 2016).

We found a heritability of 11.18% (13.87% for the moderate clusters and 10.92% for the big clusters). These heritability estimates are on the lower end of heritability estimates for in-groups, and the preference for ethnic and religious homogamy, investigated on the basis of twin data from the MIDUS data set (∼20–45%; Fieder and Huber, 2021). As we are not able to separate between additive genetics and a common (family) environment, gene–environment correlations may play an important role. Thus, additive genetics may be amplified by the common environment (parents and parental homogamy) summed up to the heritability estimate found. This is per definition a sum of genetics and family environment. Due to the correlation between genetic predisposition and the common (family) environment, there may also arise gene × environment pleiotropies as proposed by Avinun (2020). However, on the basis of our data, we are not able to detangle these effects.

As indicated, a principal limitation of our approach is that we are not able to distinguish between additive genetic effects and the effects of the parental home (common environment); thus, our estimate includes both additive genetic effects and the “common environment” (nature and nurture). In any case, using heritability calculations on the basis of a large survey, data may be a promising approach in future studies to complement analyses focusing only on the “phenotypic level.” However, this approach will need more validation on the basis of twin and future genome wide association study (GWA) heritability estimates.

Based on our data, we are not able to distinguish between the genetic and social inheritance of the predisposition of ancestral homo- or heterogamy. However, in accordance with the first law of behavioral genetics–“that all human behavioral traits are heritable”–we assume that also the tendency to marry within or outside the ancestral group should have a genetic predisposition (Turkheimer, 2000), and actually we found on the basis of the United States national survey of Midlife Development in the U.S. (MIDUS) twin sample, a preference of religious and ethnic homogamy has an additive genetic component ranging from 20 to 45% (Fieder and Huber, 2021). It has to be kept in mind that in the case of the MIDUS sample, preferences are surveyed, whereas in our study, actual marriage behavior has been investigated. Furthermore, estimates on polygenic scores indicate that actual marriage behavior may have a lower heritability (Fieder and Huber, 2021) as also indicated by the heritability results of this study.

Nevertheless, according to Fisher (1930) and Falconer (1960), also traits with a comparable low heritability can be strongly selected. Concerning selection, it is important to keep in mind, that homogamy *per se* does not change allele frequencies, but if there is an increase in the frequency of homozygous individuals, it does provide a basis on which selection may act, for instance *via* selection against the recessive homozygote (Relethford, 2012). Moreover, if the tendency to marry someone similar to one or more traits, has a genetic basis and assortative mating on this/these traits may lead to an increase in reproduction, a predisposition of assortative mating will spread in a population. However, from our findings not necessarily evolutionary assumptions may be interfered as, for instance, positive effects on fertility nowadays under a regime of birth control may differ from effects prior to birth control, this may also hold true for other changing cultural norms. Moreover, particularly for women, the “quality” of offspring (e.g., whether they are healthy and well-resourced) is also important. Hence counting children could be misleading. However, until now, the evidence that reducing the quantity for the sake of the “quality” of children and thus having more reproductive benefits, is limited, and actually, it seems that having more children in the first-generation also may lead to more children in the successive generations (Goodman et al., 2012).

We conclude that albeit only individuals of European ancestry have been included in the analysis (and irrespective of whether or not first-generation migrants are included and irrespectively of the data is aggregated), ancestry still has a relevant impact on marital and reproductive behavior. For women, the availability of a potential spouse of the according to ancestral group influences if they marry within or outside of their group, indicating a frequency depending on selection into marriage. Furthermore, the tendency of marrying within or outside the own ancestral group may also have a heritable (genetic and/or social) component; thus, selection may have been acting on this trait, albeit fitness consequences are low, but may have been higher in first- and second-generation immigrants. From a methodological perspective, we hope to encourage the analysis of heritability on the basis of large human data sets such as census data sets in the future.

These findings are also important within the context of migration and social cohesion but there is, however, a conundrum. As discussed earlier, there seems to be a tendency for homogamy, which has been demonstrated for certain traits, ancestry among them. At the same time, when considering the theory of kin-selection (Hamilton, 1964), the propensity to cooperate is related to the degree of genetic relatedness. The level of cooperation is thus highest between close relatives (e.g., parents and their children or siblings) and declines with decreasing genetic relatedness. These two divergent trends pose a dilemma: on the one side, the existing preference for homogamy, which at times has a fitness benefit (e.g., in the case of religious homogamy), and on the other side, the effects of genetic bonds ensuring the highest levels of cooperation within diverse social groups. Evidence suggests that the availability of mates within the area of residence is a decisive factor (out marriage decreases with the number of available mates of the in-group), a finding which we have also documented for religiously heterogamous marriages in Europe (Fieder et al., 2020). Therefore, as migration and admixture are continuous behavioral traits of the human species (reviewed in Reich, 2018), the dispersal of diverse populations in geographic areas will prove to be an important indicator for intermarriage rates and therefore potentially long-term social cohesion. We would emphasize more research on homogamy/heterogamy elucidating the various aspects of phenotypical and genotypical homogamy and on the reason why homogamy is persistent. Furthermore, we would like to encourage the use of the “animal model” on the basis of social science and demographic data to investigate heritability on a broader basis.

## Data Availability Statement

Only publicly available datasets were analyzed in this study. This data can be found here: https://usa.ipums.org/usa/ and can be used after regsitation at IPUMS.

## Ethics Statement

Ethical review and approval was not required for the study on human participants in accordance with the local legislation and institutional requirements. Written informed consent for participation was not required for this study in accordance with the national legislation and the institutional requirements.

## Author Contributions

AS and SH interpreted the data and wrote the manuscript. MF conducted the analyses, interpreted the data, and wrote the manuscript. All authors read and approved the final manuscript.

## Conflict of Interest

The authors declare that the research was conducted in the absence of any commercial or financial relationships that could be construed as a potential conflict of interest.

## Publisher’s Note

All claims expressed in this article are solely those of the authors and do not necessarily represent those of their affiliated organizations, or those of the publisher, the editors and the reviewers. Any product that may be evaluated in this article, or claim that may be made by its manufacturer, is not guaranteed or endorsed by the publisher.

## Footnotes

1
https://usa.ipums.org/usa/

2
https://cran.r-project.org/web/packages/QGglmm/vignettes/QGglmmHowTo.pdf

3 Fieder M. & Huber S. Religlous homogamy explains relevant ratio of the variance in reproduction.
