# Supplementary material for: Marriage in the Melting Pot: An Evolutionary Approach to European Ancestry, Homogamy, and Fertility in the United States
Source: Front Psychol. 2022 Jul 11;13:614003. doi: 10.3389/fpsyg.2022.614003 (PMC9309885; doi:10.3389/fpsyg.2022.614003)
Supplement: Supplementary file 1 [file Data_Sheet_1.PDF]

Supplement

		N	%
a) Whites	White	491760	**99.68%**
	Black/African American	326	0.07%
	American Indian or Alaska Native	714	0.14%
	Chinese	64	0.01%
	Japanese	77	0.02%
	Other Asian or Pacific Islander	286	0.06%
	Other race, nec	125	0.03%
b) Blacks	Black/African American	33352	**99.30%**
	White	167	0.50%
	American Indian or Alaska Native	27	0.08%
	Chinese	5	0.01%
	Japanese	5	0.01%
	Other Asian or Pacific Islander	23	0.07%
	Other race, nec	8	0.02%
c) American Indian or Alaska Native	American Indian or Alaska Native	1032	**56.15%**
	White	761	41.40%
	Black/African American/Negro	30	1.63%
	Chinese	1	0.05%
	Other Asian or Pacific Islander	10	0.54%
	Other race, nec	4	0.22%
d) Chinese	Chinese	1880	**94.05%**
	White	69	3.45%
	Black/African American/Negro	2	0.10%
	American Indian or Alaska Native	1	0.05%
	Japanese	15	0.75%
	Other Asian or Pacific Islander	31	1.55%
	Other race, nec	1	0.05%
e) Japanese	Japanese	2221	**62.95%**
	White	1107	31.38%
	Black/African American/Negro	73	2.07%
	American Indian or Alaska Native	2	0.06%
	Chinese	56	1.59%
	Other race, nec	6	0.17%
f) Other Asian or Pacific Islander	Other Asian or Pacific Islander	2308	**77.63%**
	White	561	18.87%
	Black/African American/Negro	43	1.45%
	American Indian or Alaska Native	2	0.07%
	Chinese	28	0.94%
	Japanese	28	0.94%
	Other race, nec	3	0.10%

Table S1) Frequency of inter- racial marriages, according to White, Black, native American, Chinese and Japanese ancestry.

code	education
0.00	N/A (or None, 1980)
1.00	None
2.00	Nursery school
3.00	Kindergarten
	Elementary school:
4.00	1st grade
5.00	2nd grade
6.00	3rd grade
7.00	4th grade
8.00	5th grade
9.00	6th grade
10.00	7th grade
11.00	8th grade
	High school:
12.00	9th grade
13.00	10th grade
14.00	11th grade
15.00	12th grade
	College:
16.00	1st year
17.00	2nd year
18.00	3rd year
19.00	4th year
20.00	5th year or more (40-50)

21.00	6th year or more (60,70)

Table S2) Encoding of highest education.

	ancestry same no yes	child born	childlessness	age	age first marriage	education	icome	income spouse	ratio ancestry county
**ancestry same no yes**	1	0.0110	0.0163	0.0159	-0.0299	-0.1592	-0.0309	-0.0839	0.3057
**child born**		1	0.8483	-0.1056	-0.2117	-0.0354	-0.0743	0.0270	0.0046
**childlessness**			1	-0.0845	-0.5232	-0.0283	-0.1105	0.0758	0.0108
**age**				1	0.1211	-0.0877	-0.0391	-0.1117	-0.0196
**age first marriage**					1	0.1712	0.0604	0.0470	-0.0738
**education**						1	0.2456	0.3300	-0.0175
**icome**							1	-0.0126	-0.0148
**income spouse**								1	-0.0385
**ratio ancestry county**									1

Table S3) Correlation matrix among the variables analysed.

**Ancestry**	**Homogamy - deviation from random mating ODDS ratios**
German	10.20
Irish, various subheads,	12.18
Scottish	12.82
English	15.24
French	18.59
Welsh	20.96
Swedish	22.92
Dutch	28.99
Danish	36.87
American Indian (all tribes)	44.42
Polish	50.08
Norwegian	56.74
Austrian	66.44
Italian	71.10
Swiss	77.73
Hungarian	78.62

Bohemian	128.28
Czechoslovakian	129.34
Lithuanian	165.92
Russian	180.26
Slovak	205.79
Romanian	298.79
Belgian	316.01
Finnish	320.17
Ukrainian	346.04
French Canadian	358.10
Croatian	463.08
Scandinavian, Nordic	541.29
Spanish	555.90
American	579.51
Canadian	600.15
Yugoslavian	802.08
Portuguese	1024.83
Greek	1125.07
Lebanese	1340.35
Mexican	2635.26
Mexican American	3981.24
White/Caucasian	4244.31
Armenian	6559.23
Puerto Rican	8279.91
Cuban	14874.98

Table S4) ODDs ratios of homogamy compared to random mating – unclustred data.

**Ancestry**	**Homogamy - deviation from random mating ODDS ratios**
German Speaking: Austria, Germany	11.12
Scandinavian: Danish, Swedish, Fin , Norwegian, Icelander	13.21
Irish	13.25
UK, British:: English, Scottish, Australian, Canada English	16.48
Dutch	33.24
Polish	55.14
Italian	79.36
Middle European Slavic: Czechoslovakian, Slovakian	100.61
French, French Canadian	205.79

Hungarian	225.71
Eastern Slavic: Ukrainian, Russian	247.44
Croatian, Slovenian	366.87
Iberian: Spanish, Portuguese, Spanish American	592.24
Greek	721.94
South Slavic, Orthodox	972.28

Table S5) ODDs ratios of homogamy compared to random mating. Moderately clustered.

Ancestry	Homogamy - deviation from random mating ODDS ratios
German Speaking: Austria, Germany	9.75
Irish	11.83
Scandinavian: Danish, Swedish, Fin , Norwegian, Icelander	11.86
UK, British: English, Scottish, Australian, Canada English	14.14
Italian	71.46

Table S6) ODDs ratios of homogamy compared to random mating. Big cluster.
